# Comparative Transcriptome Analysis of Two Rice Varieties in Response to Rice Stripe Virus and Small Brown Planthoppers during Early Interaction

**DOI:** 10.1371/journal.pone.0082126

**Published:** 2013-12-16

**Authors:** Wenjing Zheng, Li Ma, Jiaming Zhao, Zhiqiang Li, Fuyu Sun, Xiaochun Lu

**Affiliations:** 1 Liaoning Innovation Center of the Academy of Agriculture Sciences, Shenyang, People's Republic of China; 2 Plant Protection College of Shenyang Agriculture University, Shenyang, People's Republic of China; 3 Liaoning Plant Protection Institute of the Academy of Agriculture Sciences, Shenyang, People's Republic of China; Instituto de Biología Molecular y Celular de Plantas, Spain

## Abstract

Rice stripe, a virus disease, transmitted by a small brown planthopper (SBPH), has greatly reduced production of japonica rice in East Asia, especially in China. Although we have made great progress in mapping resistance genes, little is known about the mechanism of resistance.

By de novo transcriptome assembling, we gained sufficient transcript data to analyze changes in gene expression of early interaction in response to SBPH and RSV infection in rice. Respectively 648 and 937 DEGs were detected from the disease-resistant (Liaonong 979) and the susceptible (Fengjin) varieties, most of which were up-regulated. We found 37 genes related to insect resistance, which mainly included genes for jasmonate-induced protein, TIFY protein, lipoxygenase, as well as trypsin inhibitor genes and transcription factor genes. In the interaction process between RSV and rice, 87 genes were thought to be related to RSV resistance; these primarily included 12 peroxidase biosynthesis genes, 12 LRR receptor-like protein kinase genes, 6 genes coding pathogenesis-related proteins, 4 glycine-rich cell wall structural protein genes, 2 xyloglucan hydrolase genes and a cellulose synthase. The results indicate that the rice-pathogen interaction happened both in disease-resistant and susceptible varieties, and some genes related to JA biosynthesis played key roles in the interaction between SBPHs and rice. When rice was infected by RSV a hypersensitive reaction (HR) in the disease-resistant variety was suppressed, which resulted from an increase in peroxidase expression and down-regulation of LRR receptor-like protein kinase and pathogenesis-related proteins, while, the changes of peroxidase biosynthesis, glycine-rich cell wall structural protein, cellulose synthase and xyloglucan endotransglucosylase/hydrolase could lead to the strengthening of physical barriers of rice, which may be an important resistance mechanism to RSV in rice.

## Introduction

Rice stripe virus (RSV), transmitted by a small brown planthopper (SBPH, *Laodelphax striatellus* Fallen) [1], has caused a disastrous disease of rice in East Asia, particularly in China, Japan, Korea and North Korea [2]. When infected with RSV at the seeding stage, rice grows poorly and often develops folded and twisted leaves, with the central leaves yellowing and withering; growth may terminate and ultimately the plant will die. When infection occurs after tillering, upper leaves become discolored or yellow-green and the number of grains per ear is reduced significantly [3]. In China, rice stripe is increasing in severity, particularly in Jiangsu province, where approximately 0.6 M ha per year of rice were infected by RSV in the period 2000 to 2003, rising to 1 M ha in 2004. Rice yield is reduced by 30–50% in heavily infected fields, and in some of the most severely infected fields, no harvest is possible [4]. Rice stripe is still one of the most damaging virus threats to Japonica production in China. It is difficult to control this disease because the virus is transmitted in a circulative, transovarial propagative manner by viruliferous female planthoppers to their offspring. The most economical and effective way for controlling the disease is to plant resistant varieties.

The resistance gene to RSV that has been frequently utilized in breeding is Stvb^i^, which originated in the indica cultivar ‘Modan’. Hayanoo et al constructed a physical map spanning 1.8-cM distance between flanking markers, consisting of 18 bacterial artificial chromosome (BAC) clones, around the Stvb-i locus on rice chromosome 11 [5]. Despite considerable progress in mapping RSV resistance genes [6], [7], there have been only a few studies of the resistance mechanism [8], [9]

Understanding the responses of rice to RSV infection is important for developing strategies for disease control. Since rice responses to viral infection are complex and relate to many kinds of physiological processes, system-level transcriptomic studies are required to fully understand the responses. Recently, next-generation deep-sequencing techniques such as Illumina RNA-Seq and digital gene expression have provided new approaches for studying the transcriptome. RNA-Seq is a whole transcriptome sequencing method, and many transcriptome studies have greatly extended our knowledge of mechanisms of resistance to plant pathogens [10], [11], [12].

In this study, we analyzed the early response of two rice cultivars to infection by RSV and its carrier at the transcriptome level using next-generation deep-sequencing techniques. We investigated the alteration in gene expression between a disease-resistant cultivar and a susceptible cultivar before and after inoculation with RSV by co-culturing with *Laodelphax striatellus* for 48 h. Our study provides insight at the molecular level into the mechanism of development of rice stripe disease, which contributes to our understanding of the rice-RSV interaction.

## Results

### Illumina sequencing and aligning to the reference genome

**Figure 1:**
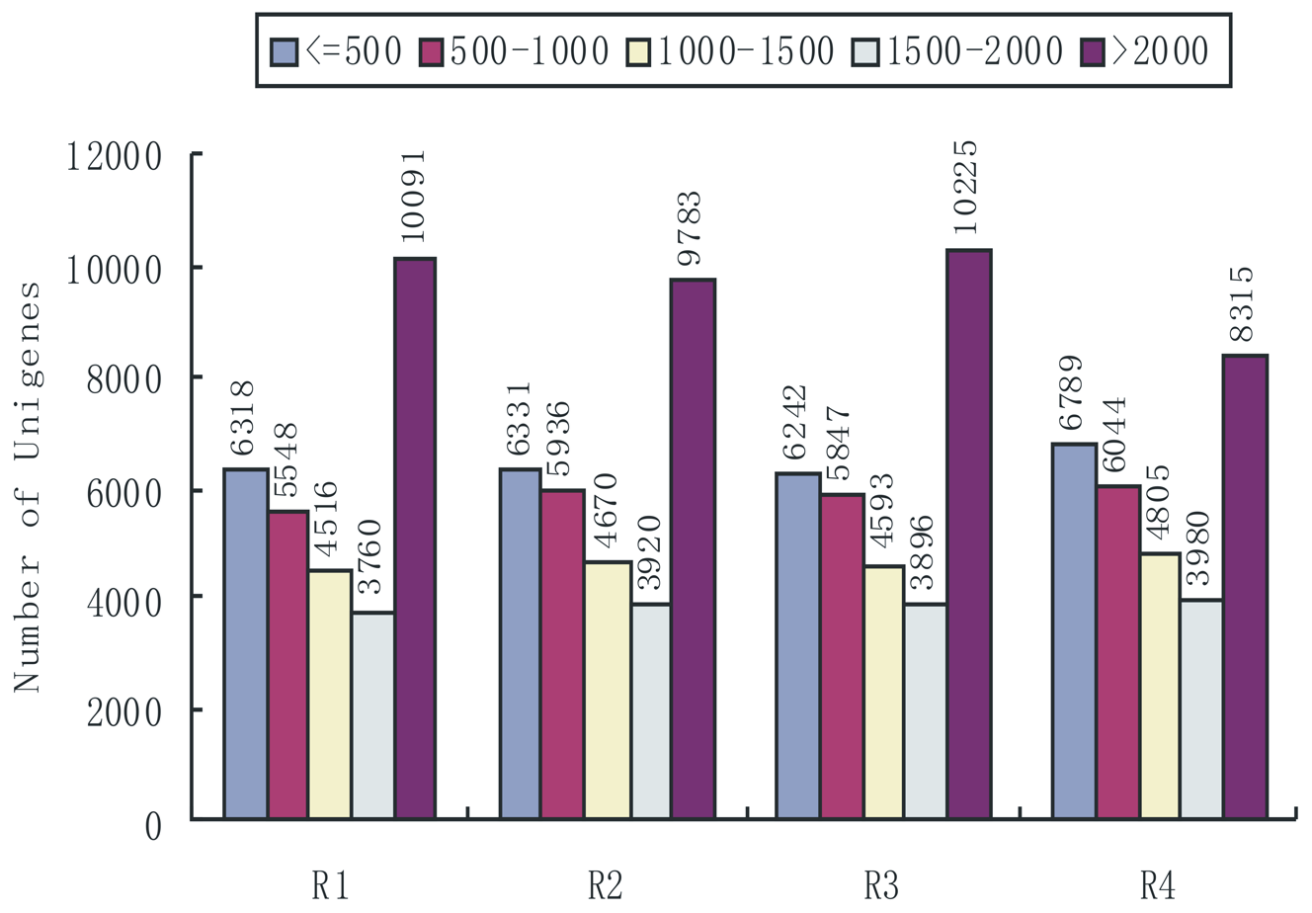
Length distribution of unigenes in the assembled transcriptomes. The x axis shows the lengths of unigenes calculated in our library and the y axis shows the number of unigenes.

Our transcriptome sequence data included gene expression profiling following different treatments of two rice varieties. The RNA-Seq method generates absolute information rather than relative gene expression measurements; thus, it avoids many of the inherent limitations of microarray analysis. This method was used to analyze differences in gene expression between the disease-resistant and the sensitive hosts. We sequenced four cDNA libraries, R1 (Liaonong 979 CK), R2 (Fengjin CK), R3 (Liaonong 979 inoculated treatment), R4 (Fengjin inoculated treatment), and generated 45,763,250 sequence reads, each of which was 50–101 bp in length, encompassing 7.91 Gb of sequence data (Table 1). GC% of sequence data from the four libraries were all approximately 50%, and CycleQ20% were all 100%, which showed that the accuracy and quality of the sequencing data were sufficient for analysis. Each treatment was represented by approximately 10 million reads, the tag density sufficient for the quantitative analysis of gene expression. Of the total reads, 70–80% matched to genomic locations. Sequence data from the four libraries were combined, and 33420 unigenes were finally obtained with an average depth of >70x and 55–60% coverage. The length distribution of total unigenes had similar patterns among the four samples, suggesting there was no bias in the construction of the cDNA libraries (Figure 1).

**Table 1:** Summary statistics for rice genes based on the RNA-Seq data.

	R1	R2	R3	R4
Total reads	10597887	10006748	12342123	12816492
sequence data	1.94Gb	1.83Gb	2.1Gb	2.04Gb
GC(%)	49.88	50.64	50.74	51.08
CycleQ20%	100	100	100	100
Mapped reads	8727756	8895279	10285064	10131598
UniqReads	7189441	7466182	8643772	8581369
MultiPosiReads	1538315	1429097	1641292	1550229
Total genes	30233	30639	30801	29928
Mean Depth	73.17	76.18	88.37	88.25
Mean Coverage	0.6	0.59	0.6	0.55

### Difference of sequencing data between the reference genome (Nipponbare) and two rice cultivars

As is shown in Table 2, many new genes, new exons,UTRs,rtIntroes, skippedexons and alternativesplices were detected from the four cDNA libraries (Table S1–6). Annotations of new genes in eight kinds of database were listed in Table S7.

**Table 2:** New information detected from the four cDNA libraries.

	R1	R2	R3	R4
Total new genes	958	963	997	1000
Total new exons	226	242	186	501
UTR	624	661	729	669
rtIntro	5461	5032	6470	5666
Skippedexon	436	585	482	1011
AlternativeSplice	13	6	9	6

**Figure 2:**
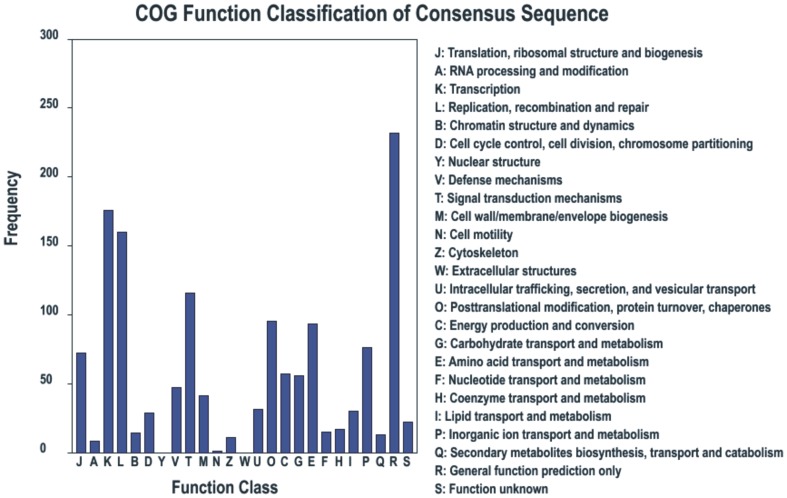
Cog function Classification of new genes. 982 new genes were categorized into 25 functional groups, the y axis shows the frequency of new genes calculated in our library and the × axis shows the activity of new genes.

We detected a total of 3918 new genes by RNA-Seq, annotated results of the different lengths of the new genes are shown in Table 2. 982 new genes were categorized into 25 functional groups with a COG classification. Among these COG categories, the cluster of ‘general function prediction’ occupied the highest number, followed by ‘transcription’, ‘replication, recombination and repair’ and ‘signal transduction mechanisms’. It's worth mentioning that 48 and 45 new genes were categorized into the defense mechanisms functional group (V) and cell wall/membrane/envelope biogenesis group (M), respectively (Figure 2). Analysis of these genes will contribute to our knowledge of the resistance mechanism to RSV and its transmission vector.

### Differentially Expressed Genes(DEGs)among the four rice samples

**Figure 3:**
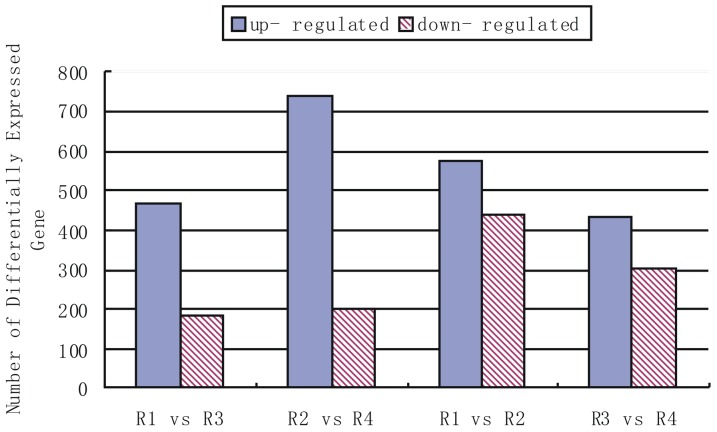
Changes in profiles of gene expression among four samples. R1  =  Liaonong 979 CK, R2  =  Fengjin CK, R3  =  Liaonong 979 inoculated treatment, R4  =  Fengjin inoculated treatment. In this graph, for example, In R1 vs R3, compared with R1, there are 475 genes up- regulated and 183 genes down- regulated in the R3 library, while in R2 vs R4 there are 737 up-regulated genes and 199 down-regulated genes in the R4 library when compared to R2. In general most of the DEGs were UP regulated whether in the resistant cultivar or in the susceptible cultivar after being inoculated by SBPH carrying RSV.

A total of 3332 DEGs were detected by RNAseq. There were 1011 differentially expressed genes between Liaonong 979 CK (R1) and Fengjin CK (R2), of which 575 genes were up-regulated and 436 genes were down-regulated. The results show that there are wide discrepancies in gene expression in seedlings of the rice cultivars. After inoculation by SBPHs that carried RSV, gene expression of the two rice varieties changed significantly. 648 differentially expressed genes were detected in Liaonong 979, among which expression levels of 465 genes were up-regulated; only 183 genes were down-regulated. In Fengjin, even though it is sensitive to RSV, 937 differentially expressed genes were detected, among which 78.7% were up-regulated. There were 736 differentially expressed genes between R3 and R4 (Figure 3).

**Figure 4:**
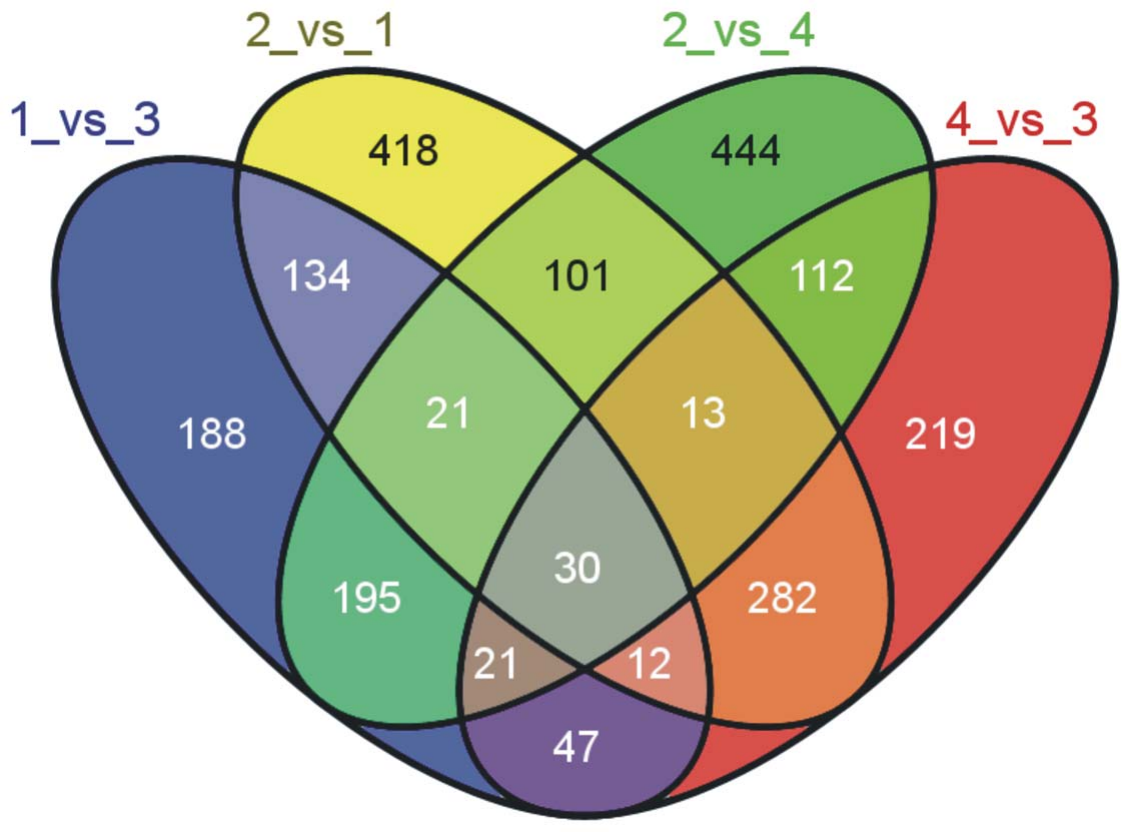
Venn diagram showing all of the DEGs.

Inoculating rice cultivars, Liaonong 979 and Fengjin, respectively resistant and sensitive to RSV, with SBPHs carried RSV, resulted in interaction with pathogens. In this process, the two cultivars shared 267 DEGs in response to piercing and sucking by SBPHs according to the Venn diagram (Figure 4). In addition, the number of DEGs between R1_vs_R3 and R2_vs_R4 was 381, and there were 399 DEGs between R3_vs_R4 and R1_vs_R2 (Table 3). These genes were predicted to be relevant to interaction between rice and SBPHs or RSV. The data illustrate the difference between the resistant and sensitive cultivars, and deep analysis of these genes may shed light on the resistance mechanism.

**Table 3:** Accounting of the DEGs shown in Figure 4.

DEGs in the Venn diagram	Function of the DEGs	Number of DEGs in the Venn diagram
Shared by 1_vs_3 and 2_vs_4	DEGs related to SBPH resistance	267(195+21+30+21)
Between 1_vs_3 and 2_vs_4	DEGs related to RSV resistance	381(188+134+47+12)
Shared by 2_vs_1 and 4_vs_3	DEGs between the two cultivars	337(282+12+30+13)
Between 2_vs_1 and 4_vs_3	DEGs related to RSV resistance	399(112+219+21+47)

### Differential gene expression profiles in response to SBPH feeding

After being inoculated by SBPHs, Liaonong 979 and Fengjin shared 267 DEGs, in which 221 genes could be annotated in the Swiss-Prot protein sequence database (Table S8). There were 27 genes that, when up-regulated were expressed in one cultivar and down regulated in the other, while the remaining genes were all either up-regulated or down-regulated in each of the two cultivars.

**Figure 5:**
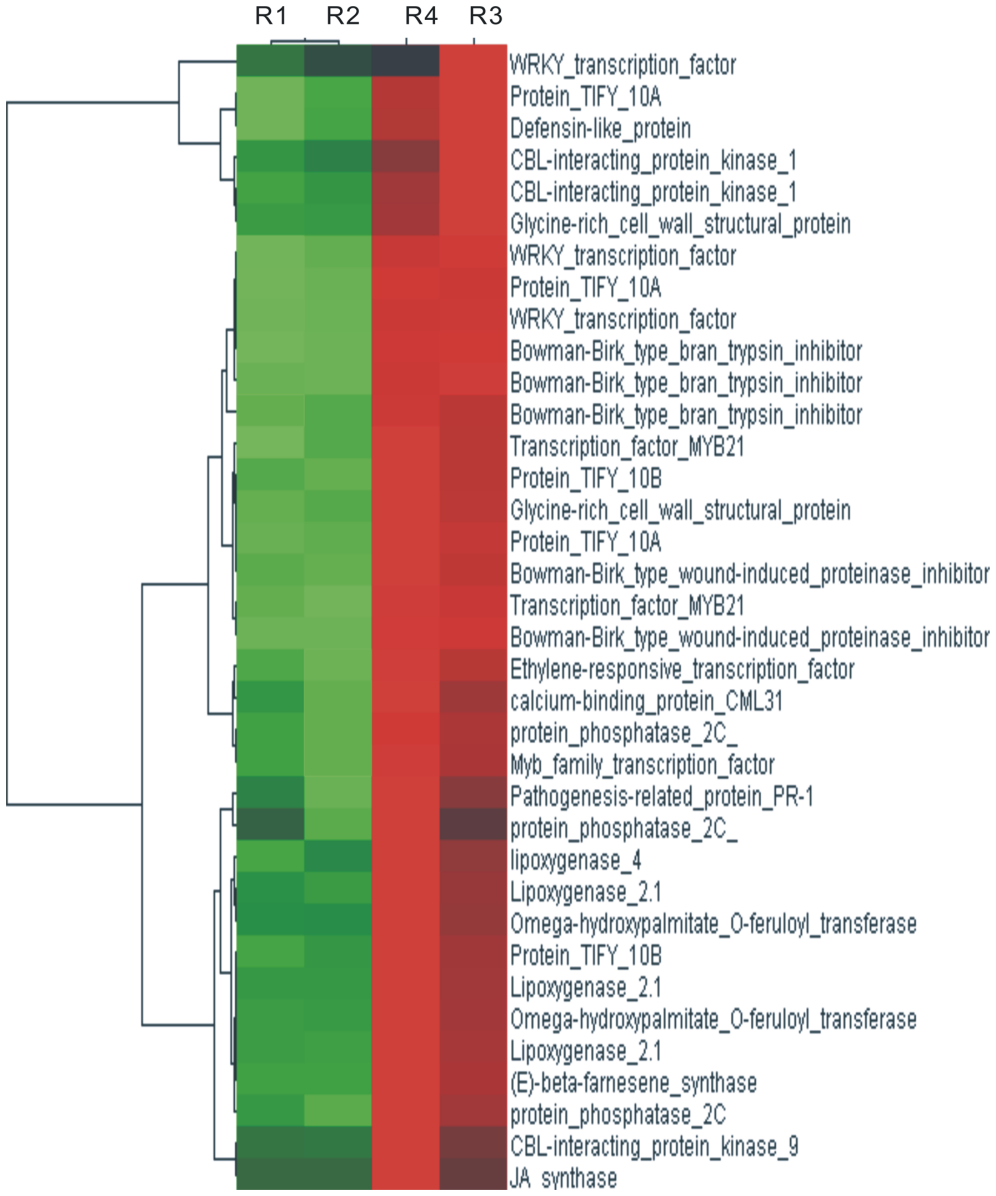
Important genes in the interaction between rice and SBPHs. From left to right, the four columns respectively show the expression of R1 (Liaonong 979 CK), R2 (Fengjin CK), R4 (Fengjin inoculated treatment) and R3 (Liaonong 979 inoculated treatment). Every row shows a different gene. Green, black and red indicate expression levels of genes, respectively low, medium and high.

Analysis of these 221 DEGs by annotation in the Swiss-Prot database showed that 37 DEGs related to interaction between rice and pathogens were all up-regulated in these two varieties. Because Liaonong 979 and Fengjin were all moderately resistant to SBPHs, the 37 DEGs having similar expression patterns in these two cultivars were considered to be related to SBPHs response. As was determined from the Swiss-Prot protein sequence database, these up-regulated DEGs included 1 jasmonate-induced protein gene, 5 TIFY protein genes, 4 lipoxygenase genes, 4 trypsin inhibitor genes, 6 transcription factor genes, 3 CBL-interacting protein kinase genes, 3 protein phosphatase 2C genes, 1 (E)-beta-farnesene synthase gene (Figure 5).

Jasmonic acid (JA) is a signal molecule extensively involved in the regulation of plant growth and response to stress [13], [14], [15]. In this study, piercing and sucking by the SBPHs induced expression of defense genes, of which JA was one of the most important factors. As is shown in Figure 5, RPKM of a gene coding jasmonate-induced protein changed most significantly, increasing from 4 to 7841 in Fengjin, and 10 to 2770 in Liaonong 979. In addition, 9 DEGs coding lipoxygenase and TIFY protein were related to JA biosynthesis. Lipoxygenase is a key enzyme of the octadecanoid pathway (also called LOX pathway) [16], [17], and the TIFY protein family is related to JAZ protein. Wounding by chewing insects resulted in the localized and systemic accumulation of high levels of proteinase inhibitor proteins in leaves of several plant families [18]. In this study, after inoculating with SBPHs, 5 genes coding proteinase inhibitors were all markedly up-regulated, indicating that proteinase inhibitors play critical roles in resistance to SBPHs resistance in addition to JA.

### Analysis of key genes participating in the early interaction between rice and RSV

**Figure 6:**
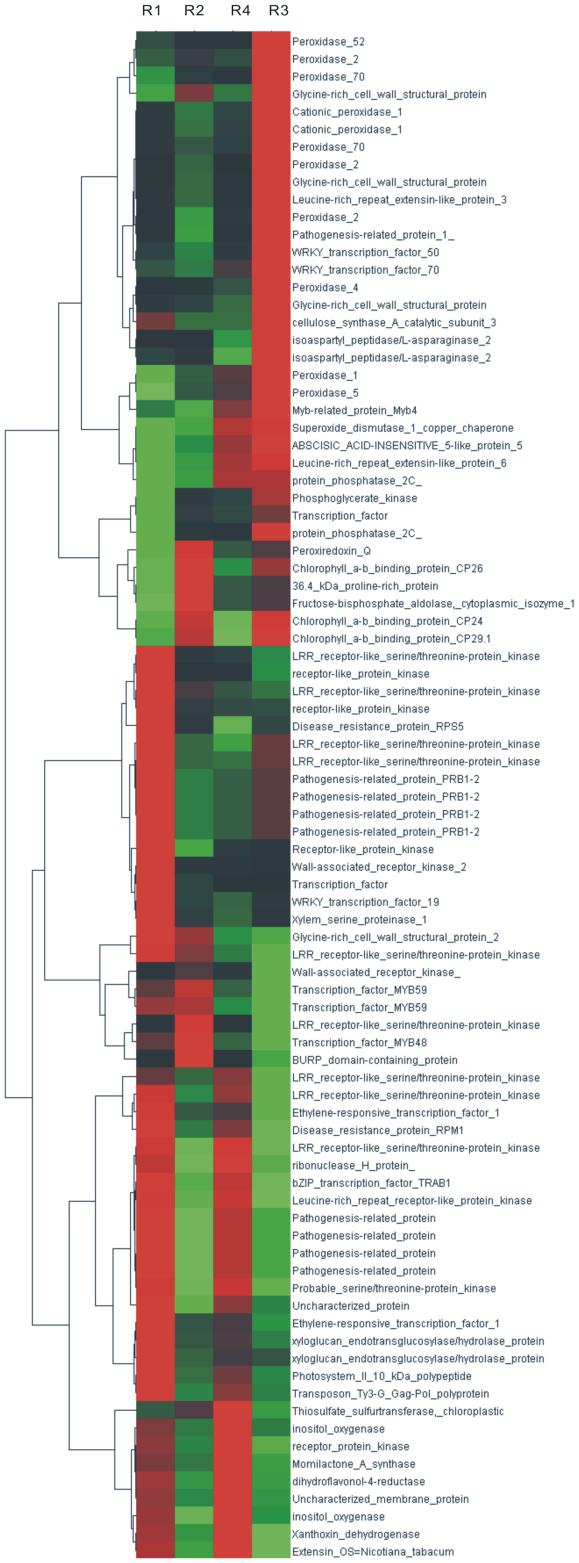
Expression patterns of some important genes related to RSV resistance. In this figure, from left to right, the four columns respectively show the expression of R1 (Liaonong 979 CK), R2 (Fengjin CK), R4 (Fengjin inoculated treatment) and R3 (Liaonong 979 inoculated treatment). Every row represents a different gene. Green, black and red indicate expression levels of genes, respectively low, medium and high.

381 DEGs were only detected in RSV resistant variety (Liaonong 979), of which 289 could be annotated in the Swiss-Prot database (Table S9). We found that 87 genes were related to plant defense responses. As is shown in Figure 6, several DEGs were significantly up-regulated; of these 12 were related to peroxidase biosynthesis, 4 to coding glycine-rich cell wall structural protein, and 1 gene coding cellulose synthase. Several other genes were markedly down-regulated, including 12 genes coding LRR receptor-like protein kinase and 6 genes coding pathogenesis-related protein, which were all related to disease resistance, signal transduction and the hypersensitive necrosis reaction (HR). Down-regulated expression of these genes showed that HR was suppressed in Liaonong 979 during the early interaction between rice and RSV. The changes in peroxidase biosynthesis, glycine-rich cell wall structural protein and cellulose synthase could result in lignification and strengthening of cell walls [19], [20], [21], which indicates that RSV resistance may be related to physical barriers of cells.

We found that expression patterns of 24 genes were different in Liaonong 979 than in Fengjin (Table S10). These genes included 4 pathogenesis-related protein genes, 2 xyloglucan hydrolase genes, 3 chlorophyll a-b binding protein genes, 1 proline-rich protein gene. The results showed that RSV infection may trigger a series of biochemical reactions, and resistance to RSV may be related to changes in photosynthesis, xyloglucan hydrolysis, and proline enrichment.

### New exons related to disease resistance

Induction of expression of 152 new exons was detected in R3 (Table S11), in which 32 and 82 new exons could be annotated respectively in COG and the Swiss-Prot databases. COG annotatation revealed 7 exons related to cell wall/membrane/envelope biogenesis. In addition, it is reported that an h-type thioredoxin functions in tobacco defense responses to two species of viruses [22], we detected a new exon encoding TPR repeat-containing thioredoxin. Whether these new exons are related to resistance to RSV is unknown.

### Comparison of DGE tag data with qRT-PCR expression patterns

**Figure 7:**
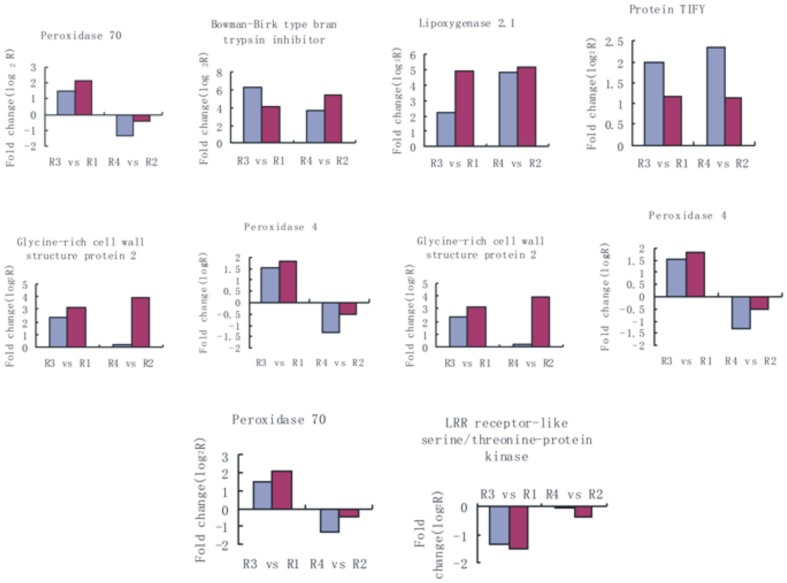
Quantitative RT-PCR (qRT-PCR) validation of the relative expression levels of transcripts selected from the DGE analysis. Expression profiles of selected genes as determined by qRT-PCR (Red) and DGE (Blue). The signal intensity of each transcript was normalized using EF1a. The y-axis shows the normalized expression level of the transcript. The ×-axis indicates the result of two groups of comparisons.

In order to validate our DGE data, ten annotated unigenes having annotations were selected for qRT-PCR analysis (Figure 7). The results showed that the qRT-PCR data of these genes were consistent with the DGE results. For example, both qRT-PCR and DGE analyses showed that genes encoding jasmonate-induced protein, Bowman-Birk type bran trypsin inhibitor, lipoxygenase 2.1 and protein TIFY were more highly expressed in two inoculated rice samples (R3, R4) than in the two non-inoculated samples (R1, R2). Likewise, expression patterns of two genes encoding peroxidase in the two rice varieties were confirmed to be similar by DGE analysis and qRT-PCR analysis. We further analyzed expression of three genes encoding LRR receptor-like serine/threonine-protein kinase and disease resistance protein and xyloglucan hydrolase protein respectively, by DGE analysis; these genes were significantly down-regulated in the resistant variety but were little changed in the susceptible variety, which is also in agreement with our qRT-PCR results.

## Discussion

### HR may not be the mechanism for resistance to RSV in Liaonong 979

Plants are often attacked by pathogens, such us bacteria, fungi, stramenopiles, viruses, protozoa and nematodes. To combat the pathogens, plants have evolved sophisticated and effective defense mechanisms. The hypersensitive response (HR) is a common initial response to attack by pathogens. This type of resistance often occurs in the incompatible interactions between plants and pathogens, and is characterized by rapid necrosis of infected and neighboring host cells [23], [24]. Previous transcriptome analysis using Illumina sequencing-based DGE led to the conclusion that HR could be considered as the mechanism of resistance to TMV and CMV [25]. However, our data indicated that HR may not be the mechanism for resistance to RSV in Liaonong 979. As can be seen in Figure 6, which shows plant disease resistance signals in the disease-resistant cultivar, the expression of 22 genes, encoding receptor-like protein kinase and pathogenesis-related protein did not increase, rather these genes were down-regulated significantly after infesting rice with SBPHs that carried RSV; Thus HR was not triggered by RSV attack in Liaonong 979.

### Lignification and cell wall strengthening may play a critical part in resistance to RSV

In addition to HR mediated by pathogenesis-related proteins, lignification was considered to be an important mechanism of resistance to pathogens in plants [26]. Lignin and callose can function as physical barriers to prevent propagation and movement of pathogens [27]. In this study, after inoculation, some DEGs including 12 peroxidase genes,4 genes coding glycine-rich cell wall structural protein and a cellulose synthase gene were markedly up-regulated in the disease-resistant host (Liaonong 979) and down-regulated or unchanged in the susceptible cultivar Fengjin. Peroxidases, glycine-rich cell wall structure protein and cellulose synthase have a role in defense by strengthening cell physical barriers and lignification, which indicates that strengthening physical barriers may be one of the factors for RSV resistance in rice. Previous studies have used different methods to characterize changes in expression of some genes in response to RSV infection. By microarray, Satoh et al studied changes in gene expression at 5 stages, respectively 0 d, 3 d, 6 d, 9d and 12d after inoculation with SBPHs carrying RSV. At 12 days post-inoculation, two families of genes, lignification and cellulose synthesis were all significantly down-regulated in Nipponbare (susceptible to RSV), which is consistent with the results in this test.

### Signal pathway induced by SBPH feeding might be similar to BPH in rice

In this study, a few genes including the jasmonate protein gene, TIFY protein genes and lipoxygenase genes were up-regulated significantly after these two rice varieties were co-cultured with SBPHs for 48 h, which indicated that the JA signaling pathway was activated by SBPH piercing and sucking. Similar results were obtained from a study of gene expression during feeding by brown planthoppers (BPH) [28]. Using microarray, expression of some genes that take part in a jasmonic acid-independent pathway were detected to be up-regulated when rice plants were co-cultured with BPH for 72 h,. Plant defense responses to attack by insects include the activation of pathways dependent on SA and JA/ethylene signaling molecules [29], [30], [31]. Results of microarray and transcriptome sequencing showed that signal pathways induced by BPH and SBPH were all JA signaling.

## Conclusion

By using next generation sequencing, abundant and high quality data were gained for the analysis of early interaction of rice in response to SBPH and RSV infection. The results indicated that expression of many genes associated with JA signaling, pathogen-related molecular patterns and cell wall structure would be activated or suppressed before and after being inoculated with SBPH carried RSV. Further analysis shows that HR may not be the mechanism for resistance to RSV in a disease resistant variety, and lignification and cell wall strengthening may play a critical part in resistance to RSV. But the molecular function of some genes remains largely unknown and requires further study. Even so, the identification of DEGs involved in rice stripe disease resistance will extend our understanding of the complex molecular and cellular events in this process and provide a foundation for future studies on breeding for resistance to rice stripe disease.

## Materials and Methods

### Plant materials

Liaonong979 is a highly resistant rice variety to RSV, and Fengjin is a highly susceptible cultivar to rice stripe disease, both of these varieties are moderately resistant to SBPH [32].

### Obtaining samples for transcriptomic analysis

Two rice cultivars, Fengjin and Liaonong979, were grown in 1000 ml beakers, 20 seeds per beaker, in a green house (25±1°C, natural sunlight). When the most vigorous seedlings reached the three-leaf stage, 100 small brown planthopper (SBPH) nymphs in the second or third instars were scattered into each beaker, 90% of which were viruliferous, and the virus-carrying rates were calculated by Enzyme-linked Immuno sorbent Assay (ELISA). SBPHs were scattered around the beaker three times daily to prevent them from remaining in one area. After 48 h, the insects were completely removed from the plants and parts of the leaves of every seedling were mixed for RNA extraction. RNA was extracted by the same method from two uninoculated controls (the control experiment without insects feeding, R1, R3).

### RNA extraction and quality determination

Total RNA was isolated using the modified CTAB method performed as described by Chang et al [33], and the RNA samples were treated with DNase I (TaKaRa, Japan) for 4 h. The integrity of the RNA samples was examined with an Agilent 2100 Bioanalyzer.

### cDNA library preparation and Illumina sequencing

cDNA library preparation and sequencing reactions were conducted by the Biomarker Technology Company, Beijing, China. Preparation of the paired-end libraries and sequencing were performed following standard Illumina methods and protocols. The cDNA library was sequenced on the Illumina Cluster Station and Illumina Genome Analyzer system.

### *De novo* assembly

Reads from each library were assembled separately. The Trinity method [34] was used for *de novo* assembly of Illumina reads of hawthorn (*Crataegus* sp.). Trinity consists of three software modules: Inchworm, Chrysalis and Butterfly, applied sequentially to process large volumes of RNA-Seq reads. In the first step in Trinity, reads are assembled into the contigs by the Inchworm program. The minimally overlapping contigs were clustered into sets of connected components by the Chrysalis program, and then the transcripts were constructed by the Butterfly program. Finally, the transcripts were clustered by similarity of correct match length beyond the 80% of longer transcripts or 90% of shorter transcripts using the multiple sequence alignment tool BLAT [35]. The raw sequence data of four samples in this test have been uploaded to NCBI(http://trace.ncbi.nlm.nih.gov/Traces/sra_sub/sub.cgi?), and the accession number is SRP028592.

### CDS analysis

The coding sequence (CDS) of the unigene was predicted by ‘getorf’ model of EMBOSS (http://emboss.sourceforge.net/apps/cvs/emboss/apps/getorf.html/), the longest CDS are recognized as the complete CDS sequence of the unigene. The complete CDS sequences were compared with the CDS sequences of Nipponbare (Os-Nipponbare-Reference-IRGSP-1.0)(http://rapdb.dna.affrc.go.jp/download/irgsp1.html).

### Functional annotation

We annotated unigenes based on a set of sequential BLAST searches [36] designed to find the most descriptive annotation for each sequence. The assembled unigenes were compared with sequences in the National Center for Biotechnology Information (NCBI) non-redundant (Nr) protein and nucleotide (Nt) databases, the Swiss-Prot protein database, the Kyoto Encyclopedia of Genes and Genomes (KEGG) pathway database, the Cluster of Orthologous Groups (COG) database, the Translated EMBL Nucleotide Sequence Database (TrEMBL) and InterPro database. The Blast2GO program[37] was used to obtain GO annotation of the unigenes. The WEGO software was then used to perform GO functional classification of all unigenes to view the distribution of gene functions.

### Digital gene expression analysis

Gene expression levels were measured in RNA-Seq (Invitrogen) analyses as numbers of reads and were normalized with RPKM [38]. IDEG6 software [39] was used to identify differentially expressed genes in pair-wise comparison, and the results of all statistical tests were corrected for multiple testing with the Benjamini–Hochberg false discovery rate (FDR<0.01). Sequences were deemed to be significantly differentially expressed if the adjusted *P* value obtained by this method was <0.001 and there was at least a two fold change (>1 or <−1 in log 2 ratio value) in RPKM between two libraries.

### Quantitative RT-PCR (qRT-PCR) analysis

To confirm the results of pyrosequencing analysis, we determined the expression levels of 10 DEGs by qRT-PCR. Total RNAs from each sample were extracted using TRIzol reagent (Invitrogen) and treated with DNase I (Invitrogen) according to the manufacturer's protocol. The concentration of each RNA sample was adjusted to 1 mg/ml with nuclease-free water and total RNA was reverse transcribed in a 20 ml reaction system using the AMV RNA PCR Kit (TaKaRa). qPR-PCR was carried out on the LightCycler 480@ II with LightCycler 480@ SYBR I Master (Roche Applied Science, Basel, Switzerland) under the following conditions: 95 μC for 5 min; and 40 cycles of 95 μC for 10 s, 60 μC for 15 s, and 72 μC for 20 s, followed by melting curve generation (68uCto95uC). Primers used in qRT-PCR for validation of differentially expressed genes were shown in Table 4.

**Table 4:** Primers used in qRT-PCR for validation of differentially expressed genes.

Gene ID	Foreword primer	Reverse primer
Os01g0124000	attgctgcgacaaggcct	cgacgacgaattcaccat
Os02g0240300	acatctacaacgacgcca	ttgtcgaacgcgttctgc
Os12g0247700	ctgttgactcaatctcgt	gatgttataacggtgctg
Os12g0559200	tggaatcatcgagagcgc	tctatcgtcagctccagc
Os08g0539700	agccttcgcaacaccaat	agaagctccaatgacgtc
Os10g0450800	tcaggctatggatctggc	gccgagtataggactagt
Os12g0611100	agttgccatcaaggtgct	gtgcaagactgttgttct
Os07g0615200	aaggtgctcgtgttcaac	tgagctgtaggcaagcta
Os06g0335900	acgtcggaccactggtac	tcagtactgcggcatgct
Os02t0240100-01	ccgcgtctacaacgacac	ccgttgaagagctcctgg

## Supporting Information

Table S1 **New genes detected from the four cDNA libraries.** These genes are those which have not been found in a japonica cultivar Nipponbare (below).
(XLS)

Table S2 **New exons detected from the four cDNA libraries.**
(XLS)

Table S3 **UTRs detected from the four cDNA libraries.**
(XLS)

Table S4 **RtIntros detected from the four cDNA libraries.**
(XLS)

Table S5 **Skippedexon detected from the four cDNA libraries.**
(XLS)

Table S6 **Alternativesplices detected from the four cDNA libraries.**
(XLS)

Table S7 **Annotation of new genes in 8 kinds of database.**
(XLS)

Table S8 **DEGs shared by liaonong 979 and Fengjin.** These genes were considered to be related to SBPH resistance.
(XLS)

Table S9 **DEGs only detected in Liaonong979.** These genes were considered to be related to RSV resistance.
(XLS)

Table S10 **24 DEGs whose expression patterns were adverse in Liaonong979 and Fengjin.** These genes were considered to be related to RSV resistance.
(XLS)

Table S11 **152 Induced expressing new exons detected in R3.**
(XLS)

## References

[1] Kuribayashi K (1931) On the relationship between rice stripe disease and *Delphacodes striatella* Fallen. J Plant Prot (Tokyo) 18: : 565–571,636–640.

[2] Toriyama S (1983) Rice stripe virus. CMI/ABB. Description of plant viruses 269: 15–16.

[3] Kiso A, Yamamoto T (1973) Infection and symptom in rice stripe disease with special reference to disease-specific protein other than virus. Rev Plant Prot Res 6: 75–100.

[4] Sun DZ, Jiang L (2006) Research on the inheritance and breeding of rice stripe resistance. Chin Agric Sci Bull 22: 318–322 (in Chinese with English abstract).

[5] Hayano-Saito Y, Satio K, Nakamura S, Kawasaki S (2000) Fine physical mapping of the rice stripe resistance gene locus, Stv-bi. Theoret Appl Genet 101: 59–63.

[6] Zhang YX, Wang Q, Jiang L, Liu LL, Wang BX, et al (2011) Fine mapping of *qSTV11*(KAS), a major QTL for rice stripe disease resistance. Theor Appl Genet 122: 1591–1604. 21384112 10.1007/s00122-011-1557-0 PMC3082044

[7] Wang Q, Liu Y, Hu J, Zhang Y, Xie K, et al (2013) Detection of Quantitative Trait Loci (QTLs) for Resistances to Small Brown Planthopper and Rice Stripe Virus in Rice Using Recombinant Inbred Lines. Int J Mol Sci 14: 8406–8421. 23591851 10.3390/ijms14048406 PMC3645751

[8] Satoh K, Kondoh H, Sasaya T, Shimizu T, Choi IR, et al (2010) Selective modification of rice (*Oryza sativa*) gene expression by rice stripe virus infection. J Gen Virol 91: 294–305. 19793907 10.1099/vir.0.015990-0

[9] Hao Z, Wang L, He Y, Liang J, Tao R (2011) Expression of defense genes and activities of antioxidant enzymes in rice resistance to rice stripe virus and small brown planthopper. Plant Physiol Biochem 49: 744–751. 21300551 10.1016/j.plaphy.2011.01.014

[10] Ishihara T, Mitsuhara I, Takahashi H, Nakaho K (2012) Transcriptome analysis of quantitative resistance-specific response upon *Ralstonia solanacearum* infection in tomato. PLoS One 7: e46763. 23071630 10.1371/journal.pone.0046763 PMC3465262

[11] Savory EA, Adhikari BN, Hamilton JP, Vaillancourt B, Buell CR, et al (2012) mRNA-Seq analysis of the *Pseudoperonospora cubensis* transcriptome during cucumber (*Cucumis sativus* L.) infection. PLoS One 7: e35796. 22545137 10.1371/journal.pone.0035796 PMC3335787

[12] Lu J, Du ZX, Kong J, Chen LN, Qiu YH, et al (2012) Transcriptome analysis of *Nicotiana tabacum* infected by Cucumber mosaic virus during systemic symptom development. PLoS One 7: e43447. 22952684 10.1371/journal.pone.0043447 PMC3429483

[13] Creelman RA, Mullet JE (1995) Jasmonic acid distribution and action in plants: regulation during development and response to biotic and abiotic stress. Proc Natl Acad Sci U S A 92: 4114–4119. 11607536 10.1073/pnas.92.10.4114 PMC41895

[14] McConn M, Creelman RA, Bell E, Mullet JE, Browse J (1997) Jasmonate is essential for insect defense in *Arabidopsis* . Proc Natl Acad Sci U S A 94: 5473–5477. 11038546 10.1073/pnas.94.10.5473 PMC24703

[15] Staswick PE (2008) JAZing up jasmonate signaling. Trends Plant Sci 13: 66–71. 18261950 10.1016/j.tplants.2007.11.011

[16] Chung HS, Howe GA (2009) A critical role for the TIFY motif in repression of jasmonate signaling by a stabilized splice variant of the JASMONATE ZIM-domain protein JAZ10 in *Arabidopsis* . Plant Cell 21: 131–145. 19151223 10.1105/tpc.108.064097 PMC2648087

[17] Kollarova R, Oblozinsky M, Kovacikova V (2013) [Physiological aspects of lipoxygenase in plant signaling systems part I. Octadecanoid pathway]. Ceska Slov Farm 62: 59–64. 23822569

[18] Farmer EE, Johnson RR, Ryan CA (1992) Regulation of expression of proteinase inhibitor genes by methyl jasmonate and jasmonic Acid. Plant Physiol 98: 995–1002. 16668777 10.1104/pp.98.3.995 PMC1080300

[19] Vance CP, Kirk TK, Sherwood RT (1980) Lignification as a mechanism of disease resistance. Annu Rev Phytopathol 18: 259–288.

[20] Ye XS, Pan SQ, Kuc J (1990) Activity, Isozyme Pattern, and Cellular Localization of Peroxidase as Related to Systemic Resistance of Tobacco to Blue Mold (*Peronospora tabacina*) and to Tobacco Mosaic Virus. Physiology and Biochemistry 80: 1295–1299.

[21] Kristensen BK, Brandt J, Bojsen K, Thordal-Christensen H, Kerby KB, et al (1997) Expression of a defence-related intercellular barley peroxidase in transgenic tobacco. Plant Sci 122: 173–182.

[22] Sun L, Ren H, Liu R, Li B, Wu T, et al (2010) An h-type thioredoxin functions in tobacco defense responses to two species of viruses and an abiotic oxidative stress. Mol Plant Microbe Interact 23: 1470–1485. 20923353 10.1094/MPMI-01-10-0029

[23] Bowles DJ (1990) Defense-related proteins in higher plants. Annu Rev Biochem 59: 873–907. 2197993 10.1146/annurev.bi.59.070190.004301

[24] Shirasu K, Schulze-Lefert P (2000) Regulators of cell death in disease resistance. Plant Mol Biol 44: 371–385. 11199395 10.1023/a:1026552827716

[25] Zhang Y, Pei X, Zhang C, Lu Z, Wang Z, et al (2012) De novo foliar transcriptome of *Chenopodium amaranticolor* and analysis of its gene expression during virus-induced hypersensitive response. PLoS One 7: e45953. 23029338 10.1371/journal.pone.0045953 PMC3461033

[26] Conti GG, Bassi M, Carminucci D, Gatti L, Bocci AM (1990) Preinoculation with tobacco necrosis virus enhances peroxidase activity and lignification in cucumber. as a resistance response to *Sphaerotheca fuliginea* . Phytopathology 128: 191–202.

[27] Iglesias VA, Meins F Jr (2000) Movement of plant viruses is delayed in a beta-1,3-glucanase-deficient mutant showing a reduced plasmodesmatal size exclusion limit and enhanced callose deposition. Plant J 21: 157–166. 10743656 10.1046/j.1365-313x.2000.00658.x

[28] Zhang F, Zhu L, He G (2004) Differential gene expression in response to brown planthopper feeding in rice. J Plant Physiol 161: 53–62. 15002664 10.1078/0176-1617-01179

[29] Li Q, Xie QG, Smith-Becker J, Navarre DA, Kaloshian I (2006) Mi-1-Mediated aphid resistance involves salicylic acid and mitogen-activated protein kinase signaling cascades. Mol Plant Microbe Interact 19: 655–664. 16776299 10.1094/MPMI-19-0655

[30] Pieterse CM, Leon-Reyes A, Van der Ent S, Van Wees SC (2009) Networking by small-molecule hormones in plant immunity. Nat Chem Biol 5: 308–316. 19377457 10.1038/nchembio.164

[31] Zarate SI, Kempema LA, Walling LL (2007) Silverleaf whitefly induces salicylic acid defenses and suppresses effectual jasmonic acid defenses. Plant Physiol 143: 866–875. 17189328 10.1104/pp.106.090035 PMC1803729

[32] Li Z-Q, Yu F-q, Shao L-y, Chen D-h, Sun F-y (2012) Rice stripe disease resistance evaluation to rice varieties of Liaoning province in field. Liaoning Agric Sci 6: 16–18 (In Chinese).

[33] Zhang CZ, Yang H, Li H, Dai H (2007) Detection of Strawberry RNA and DNA Viruses by RT-PCR Using Total Nucleic Acid as a Template. Phytopathology 155: 431–436.

[34] Grabherr MG, Haas BJ, Yassour M, Levin JZ, Thompson DA, et al (2011) Full-length transcriptome assembly from RNA-Seq data without a reference genome. Nat Biotechnol 29: 644–652. 21572440 10.1038/nbt.1883 PMC3571712

[35] Kent WJ (2002) BLAT–the BLAST-like alignment tool. Genome Res 12: 656–664. 11932250 10.1101/gr.229202 PMC187518

[36] Altschul SF, Madden TL, Schaffer AA, Zhang J, Zhang Z, et al (1997) Gapped BLAST and PSI-BLAST: a new generation of protein database search programs. Nucleic Acids Res 25: 3389–3402. 9254694 10.1093/nar/25.17.3389 PMC146917

[37] Conesa A, Gotz S, Garcia-Gomez JM, Terol J, Talon M, et al (2005) Blast2GO: a universal tool for annotation, visualization and analysis in functional genomics research. Bioinformatics 21: 3674–3676. 16081474 10.1093/bioinformatics/bti610

[38] Mortazavi A, Williams BA, McCue K, Schaeffer L, Wold B (2008) Mapping and quantifying mammalian transcriptomes by RNA-Seq. Nat Methods 5: 621–628. 18516045 10.1038/nmeth.1226 PMC13303166

[39] Romualdi C, Bortoluzzi S, D'Alessi F, Danieli GA (2003) IDEG6: a web tool for detection of differentially expressed genes in multiple tag sampling experiments. Physiol Genomics 12: 159–162. 12429865 10.1152/physiolgenomics.00096.2002

